# Mechanochemical Synthesis and Physicochemical Characterization of Isoniazid and Pyrazinamide Co-crystals With Glutaric Acid

**DOI:** 10.3389/fchem.2020.595908

**Published:** 2020-11-16

**Authors:** Jean Baptiste Ngilirabanga, Marique Aucamp, Paulo Pires Rosa, Halima Samsodien

**Affiliations:** ^1^School of Pharmacy, University of the Western Cape, Cape Town, South Africa; ^2^Faculty of Pharmaceutical Sciences, State University of Campinas, Saö Paulo, Brazil

**Keywords:** antitubercular, co-crystallization, mechanochemistry, solid-state grinding, liquid-assisted grinding, characterization, saturation solubility

## Abstract

The present work reports two novel pharmaceutical co-crystals; 2:1 isoniazid-glutaric acid (INHGA) and 2:1 pyrazinamide-glutaric acid (PGA). Isoniazid and pyrazinamide are key first-line drugs used for the treatment of tuberculosis. The co-crystals were produced *via* solid-state and solvent assisted grinding methods. Thermal characteristics of the samples were obtained using the differential scanning calorimetry, hot stage microscopy, and thermogravimetric analyses. The morphology of the powder samples by scanning electron microscopy, structural analysis by Fourier transform infrared spectroscopy and powder X-rays diffraction ensured co-crystal formation. Thermal analyses confirmed the co-crystals with new melting transitions ranging between their respective starting materials. Unique morphologies of the co-crystal particles were clear in SEM micrographs. The formation of intermolecular interactions with the co-crystal former was confirmed by the FT-IR spectral band shifting and was supported by distinct PXRD patterns of co-crystals thereby authenticating the successful co-crystal formation. *In vitro* solubility evaluation of the synthesized co-crystals by HPLC suggested a remarkable increase in solubility of both INH and PZA in their respective co-crystals.

## Introduction

For centuries, infectious diseases have been the leading cause of death in human existence. Among known infectious diseases, tuberculosis (TB), an infection caused by *Mycobacterium tuberculosis*, and HIV/AIDS caused by the human immunodeficiency virus (HIV) holds the leading rates on infection (Raviglione and Sulis, 2016) with TB ranked the second killer disease worldwide after HIV. TB is also known as the oldest infectious disease in history (Pinheiro et al., 2014), yet still a danger which the battle against is far from over. Compared to the 2012–2013 WHO report, approximately 8.6 million TB infections were recorded and 1.3 million deaths associated with TB, 10 million new cases, and 1.5 million deaths were reported in 2018. Nonetheless, 58 million lives have been saved over the period of 10 years (2010–2018) using the current TB treatment strategies (Baddeley et al., 2013).

It is estimated that one-fourth of the population worldwide are infected by latent *Mycobacterium tuberculosis* (Mtb). This serves as a reservoir for the active and deadly form of the infection, making it even harder to control the infection. The threat is further aggravated by the appearance of drug resistance making treatment very complicated and in some cases resulting in treatment failure (Somoskovi et al., 2001; Hu et al., 2017; Howard et al., 2018) and co-infection with HIV/AIDS (Pawlowski et al., 2012; Mesfin et al., 2014; Bell and Noursadeghi, 2018; Duarte et al., 2018; Letang et al., 2020).

Despite the advent of drug resistance and several other drawbacks and inconveniences for patients, the fact remains that antibiotics have remarkably contributed to the control of TB and other various infections, saving many lives by reducing the associated morbidity and mortality rate of bacterial infections (Aminov, 2010).

As anti-tubercular drugs, isoniazid, chemically known as isonicotinic acid hydrazide (INH) and pyrazinamide known as pyrazinecarboxamide (PZA) are two key drugs in the first-line treatment of TB. Both drugs are used in a single combined dose concomitant with rifampicin (RIF) and ethambutol (ETB) or streptomycin. INH and PZA are prodrugs in nature; both activated after administration, the process triggered by the bacterial enzymes. The mechanisms of action were thoroughly explained by Unissa et al. (2016), and INH is the most used drug in TB treatment. Its potential is well-understood not only by its activity but also through the meaning of multidrug-resistance TB (MDR-TB).

However, despite its remarkable activity which seems to relate to molecular targets of the prokaryotic cells, toxicity and different side effects (dryness of the mouth, flu-like syndrome, allergic reactions, peripheral neuritis, mental abnormalities, methaemoglobinemia, and hepatotoxicity), have been linked to the use of INH (Sarceviča et al., 2016).

PZA is also a very effective antimycobacterial agent that offers to the combinations its unique sterilizing activity against persistent tubercle bacilli during the initial intensive phase of treatment. The use of PZA is also associated with shortening the duration of TB treatment, from 9 to 6 months (Somoskovi et al., 2001; Chiş et al., 2005). PZA shows prodigious but pH-dependent *in-vivo* activity. It is effective against *M. tuberculosis* only under acidic conditions. However, like many other drugs, different side effects have been attributed to the prolonged use of PZA (Schaberg et al., 1996).

Regardless of their substantial contribution, there is evidence of different strains being resistant to all available antitubercular drugs as well as different inconveniences (side effects) to patients caused by the use of high doses required to achieve a better therapeutic effect. Additionally, while INH and RIF are essential in the TB treatment regimen, these two drugs present interaction issues, and the presence of PZA catalyzes their interactions. Such interactions were also reported during the use of the fixed-dose combinations containing these three substances with ETB (Battini et al., 2018). Such interactions may lead to the reduced activity of the dose, treatment failure, and the appearance of drug resistance or even advert effects (Scripture and Figg, 2006; Palleria et al., 2013; Michael et al., 2016).

Due to these setbacks, there is an intense need for new drugs and novel treatment strategies to improve the treatment of the infection. The possibility of enhancing the efficacy of existing drugs is considered a cost-effective approach to address the mentioned challenges (Aitipamula et al., 2013). Today, co-crystallization is one of the strategies used to fine-tune the physicochemical properties of drugs (Patel et al., 2012; Gadade and Pekamwar, 2016). Co-crystallization uses principles of crystal engineering to modify the physicochemical properties of the active pharmaceutical ingredients (APIs) while maintaining intrinsic drug activity (Duggirala et al., 2016). Based on this and the fact that a wider range of compounds readily available to be explored, co-crystallization has become a preferred approach over salt formation, amorphous forms, hydrates/solvates and polymorphism.

Pharmaceutical co-crystals which consist of an API and a pharmaceutically regarded as safe compound bind together in the crystal lattice (in a very defined stoichiometry), through intermolecular (non-covalent) interactions among which hydrogen bonding being the most encountered bonding type (Sekhon, 2009; Gadade and Pekamwar, 2016; Masodkar et al., 2018). Different pharmaceutical co-crystals have been reported. These include that of indomethacin-saccharin (Chun et al., 2013), pyrazinamide-diflunisal (Évora et al., 2011), isoniazid-4-aminosalicylic acid co-crystal (Grobelny et al., 2011), dimethylglyoxime with N-heterocyclic aromatic compounds and acetamide (Abidi et al., 2017), etc.

Grinding crystalline materials also known as mechanochemistry has become a method of choice for co-crystal preparation (Shan and Zaworotko, 2008; Brown, 2012; Lin, 2016). In this work, mechanochemical reaction as a co-crystal formation approach, was used to produce two novel supramolecular co-crystals of the two antitubercular drugs INH and PZA with glutaric acid (GA). Both drugs have shown the affinity to form co-crystals with carboxylic acids. Some of the co-crystals have been reported (Grobelny et al., 2011; Cherukuvada and Nangia, 2012; Luo and Sun, 2013; Diniz et al., 2018). Two of the PZA polymorphic co-crystals with dicarboxylic acids were previously reported and were synthesized *via* solution crystallization, and their structures were solved (Aitipamula et al., 2014). Other co-crystal polymorphs were listed by Gadade and Pekamwar (2016).

## Materials and Methodology

### Materials

**Figure 1:**
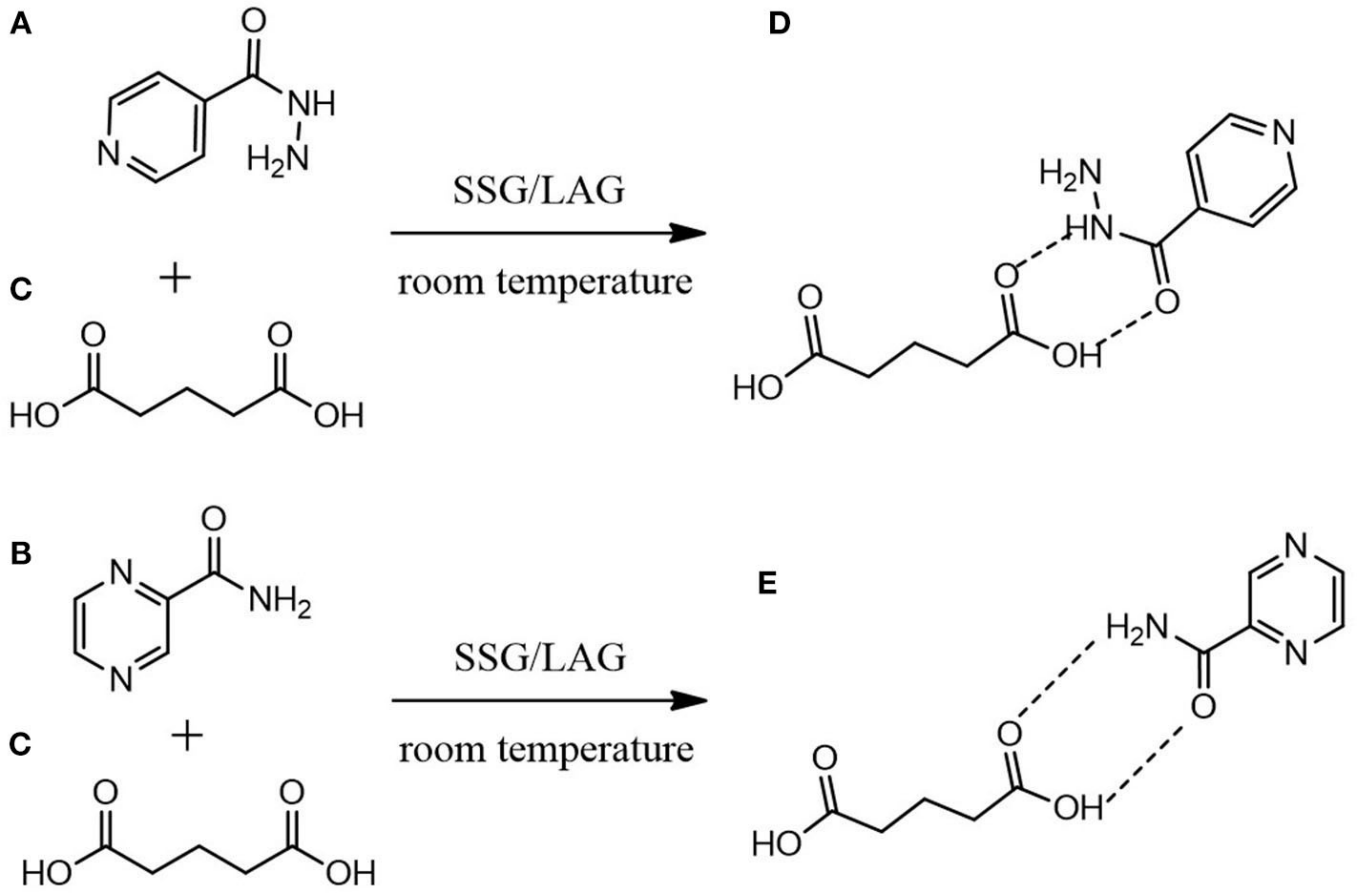
Proposed molecular mechanistic for the formation of **(D)** INHGA (2:1) and **(E)** PGA (2:1) co-crystals of **(A)** isoniazid and **(B)** pyrazinamide with **(C)** glutaric acid as a co-former using solid-state grinding (SSG) and liquid-assisted grinding (LAG).

All materials used in this project were of analytical grade. Isoniazid (purity ≥ 99.6%) and pyrazinamide (purity ≥ 98.6%) and glutaric acid (purity ≥ 99.9%) (structures depicted in Figure 1). Solvents used were purchased from Sigma-Aldrich Chemie GmbH (Steinheim, Germany) and were used as received unless otherwise stated.

### Synthesis

Both 2:1 co-crystals INHGA (2:1) and PGA (2:1) were synthesized using solid-state grinding and solvent-assisted grinding processes. Initially, the screening for the co-crystal was carried out using solid-state grinding, a method during which the mixtures INH:GA and PZA:GA in different molar ratios (1:1; 2:1; 1:2) were subjected to milling using a mortar and pestle. Both co-crystals were reproduced by the milling process assisted by a few drops of the solvent (ethanol 96%) added to the mixtures throughout the grinding process. The reproducibility of samples was ensured by repeating experiments in triplicate.

### Physicochemical Characterization

Pure APIs, co-former and the co-crystal samples were characterized by hot stage microscopy (HSM), differential scanning calorimetry (DSC), thermogravimetric analysis (TGA), Fourier transform infrared spectroscopy (FT-IR), scanning electron microscopy (SEM), and powder X-ray diffraction (PXRD). All analyses were performed in triplets.

#### Hot Stage Microscopy (HSM)

Thermo-microscopic analyses were carried out on an optical microscope Olympus (SZX-ILLB200) equipped with a Linkam temperature-controlled stage (THMS600/720), connected to a T95-PE system controller (Linkam Scientific Instruments Ltd., Tadworth, Surrey, UK). The hot-stage was calibrated using USP melting point standards. Images were recorded with an Olympus UC30 camera attached to the specified microscope (Olympus Optical, Japan) using Stream Essentials software. Samples were submerged *in silicon* oil and subjected to heating over a temperature range of 30–350°C at a constant heating rate of 10°C/min.

#### Differential Scanning Calorimetry (DSC)

The DSC measurements were carried out on a DSC 8000 Perkin Elmer instrument (Waltham, USA), incorporating a cooling system and a Perkin Elmer nitrogen gas generator. Samples (2.5–5 mg) were crimped in the aluminum pans sealed with pierced lids. The instrument was calibrated by measuring the onset temperatures of the melting of indium (m.p. 156.6°C) and zinc (m.p. 419.5°C) while the heat flow was calibrated from the enthalpy of melting of indium (28.62 J/g). The samples were heated to 20–30°C above the melting range of the sample's highest melting component, at a heating rate of 10°C/min. All analyses were performed under continuous 99.8% dry nitrogen purging with a flow rate of 20 mL/min.

#### Thermogravimetry (TGA)

A Perkin-Elmer 4000 PC thermal system (Waltham, USA) was used. The calibration of the instrument was performed using three different references; alumel (m.p. = 154.2°C), Perk alloy (m.p. = 596°C) and iron (m.p. = 780°C) at 1 and 2°C/min. The programmed TG technique was carried out over a temperature range between 30–400°C at a predetermined heating rate of 10°C/min. Samples were continuously purged by a stream of 99.8% dry nitrogen gas (20 mL/min) and solvent stoichiometry of the compounds was determined from the percentage mass loss.

#### Fourier Transform Infrared Spectrophotometry (FT-IR)

Infra-red spectra of the individual drugs and the co-crystal samples were obtained using a Perkin-Elmer 100 FTIR instrument (Waltham, USA) fitted with UATR® and controlled with Spectrum software version 6.3.5.0176 for the analysis. Analyses of powder or crystalline samples were done over the range of 650–4,000 cm^−1^ at a 2 cm^−1^ resolution.

#### Scanning Electron Microscopy (SEM)

The morphology of untreated INH, PZA, GA, and co-crystal samples were conducted using an AURIGA Field Emission High-Resolution Scanning Electron Microscope (HRSEM), Zeiss (Germany). Powder samples were mounted onto aluminum stubs using carbon tape. Accelerating voltage of 5 kV was used for images and 20 kV for EDS. The lament current was set at 2,359 amps. Carbon tabs were placed on the aluminum stubs to hold samples. Working distance (WD) for images is displayed on each image. SmartSEM 90 and AZTEC software were used for imaging and EDS, respectively.

#### Powder X-Ray Diffraction (PXRD)

PXRD data were recorded on a D8 Advance powder X-ray diffractometer (Bruker AXS GmbH, Germany) with CuKα radiation (Cu-Kα_1_= 1.54056 Å). The tube voltage and current applied were 35 kV and 40 mA, respectively. A V20 variable slit was used. Samples were placed on the sample holder which has 1 mm thickness and 1.5 cm in diameter. With a scan rate of 2° per min, sample scan was performed in a continuous scan in locked coupled mode, within a range of 2θ 5 to 50°. Origin Pro 8.5. was used to plot PXRD patterns.

#### *In vitro* Solubility Studies

Excess amounts of the individual drugs INH, PZA, and their respective co-crystals were added to USP aqueous buffer solutions at pH 1.2 (0.1N hydrochloric acid), 0.2M potassium phosphate buffer (pH 6.8 and pH 7.4), and deionized water (18.s MΩ). The mixtures were continuously shaken in an incubator shaker at 37°C (± 0.5°C) and at 100 rpm for 24 h. Samples were filtered using 0.20 μm PVDF syringe filters and analyzed using an HPLC. INH and PZA in both co-crystal preparations were quantified using an Agilent HPLC system setting with FXB Pump (Flexar Binary Pump), an automated injector equipped with a UV detector (LC 200a Series PDA Detector), and a Flexar autosampler. Isocratic elution using acetonitrile: water mixed in 30:70 (v/v) solution was applied at a flow rate of 10 mL/min. A reversed-phase Luna C_18_ HPLC column 250 mm × 4.6 mm, 5μm was used. Injection volume was 10 μL and absorbance of elution was recorded at 262 and 270 nm following suitable dilutions.

## Results and Discussion

**Figure 2:**
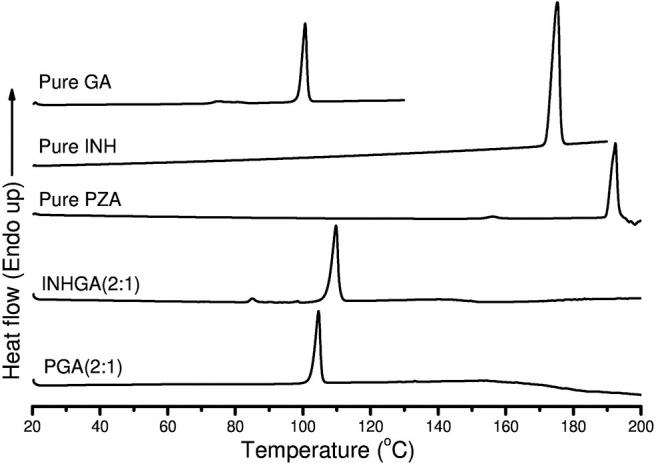
The DSC curves of pure pentanedioic acid (GA), isoniazid (INH), pyrazinamide (PZA), and two potential co-crystals INHGA (2:1) and PGA (2:1).

Thermal analysis by DSC confirmed the melting onsets of the individual drugs INH, PZA, and the co-former GA, respectively at 172.0, 189.7, and 98.6°C. The GA DSC curve showed an additional broad peak at 74.0°C corresponding to the polymorphic transition (polymorph-β to α) (Bruni et al., 2013). The 2:1 co-crystal of isoniazid-glutaric acid (INHGA) exhibited a single sharp endothermic melt at 106.9°C (Figure 2). This melting temperature falls in the range between then INH and GA melting points.

The melting onset of the 2:1 pyrazinamide-glutaric acid (PGA) co-crystal obtained from dry and/or solvent assisted co-grinding, was found at 102.4°C. The melting point ranges between melting temperatures of the PZA and co-former GA. Table 1 summarizes all the essential thermal properties of the samples.

**Table 1:** Thermal properties for pure INH, PZA, GA, and the two co-crystals INHGA (2:1) and PGA (2:1) from DSC analyses.

**Sample**	**Melting onset temperature (^**°**^C)**	**Δ_fus_*H* (J/g)**
INH	172.0	206.704
INHGA (2:1)	106.9	99.451
GA	98.6	96.711
PGA (2:1)	102.4	99.889
PZA	189.7	227.915

These unique melting points were the first indication of the successful co-crystallization experiments, although further confirmation by other analytical techniques is warranted since the change in melting points among multicomponent solid forms may occur either due to formation of non-covalent bonding interaction between API and co-formers, alteration in packing arrangements (e.g., polymorphic transition, the formation of eutectic solid mixtures or solid solution). The fact that a single peak was established during DSC analysis certainly identifies an interaction between the two parent compounds.

HSM and TGA experiments were carried out to further understand the thermal behavior of the co-crystal samples. The results obtained were subsequently compared to the DSC data. In both cases, the co-crystals remain stable until melting temperatures are reached. Results obtained from HSM (Supplementary Figure 2) and TGA analyses showed no thermal changes before melting occurs and correlate with DSC outcomes. Based on the HSM analysis, GA melted between 98 and 101°C range. Co-crystals INHGA (2:1) and PGA (2:1) showed a melt in the temperature ranges 106–111and 102–108°C, respectively, thus matching the DSC results.

**Figure 3:**
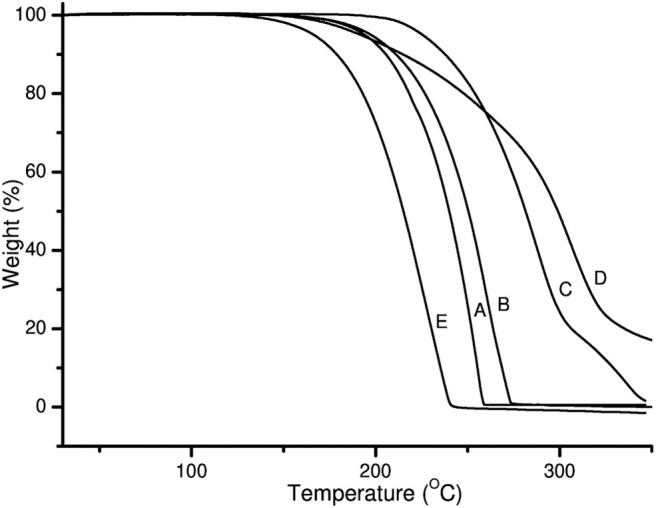
TGA thermograms of (A) PZA, (B) GA, (C) INH, (D) INHGA (2:1) and (E) PGA (2:1) co-crystal.

TGA thermograms of pure PZA, GA, INH, and both co-crystals are shown in Figure 3. The TGA thermogram generated from pure PZA showed a stepwise mass loss with initial 24.8% loss occurring before and over melting range (from 150 to 205°C), suggesting the presence of moisture in PZA sample. The second mass loss occurred due to the decomposition of the sample over 205–245°C temperature range with 74.1% of mass loss (Figure 3A). GA generated a TGA thermogram (Figure 3C) showing only a decomposition mass loss over 150–250°C.

On the other hand, the TGA thermogram of pure INH exhibited a stepwise mass loss with decomposition (onset at 229°C). The first step indicated 81.3% loss of the initial sample weight followed by 18% loss (Figure 3C).

The mass loss recorded by TGA prior to the melting range was insignificant (0.0%) for both co-crystals INHGA (2:1), and PGA (2:1), thus confirming that they are anhydrous and solvent-free. The TGA curves are further characterized by single steps of weight loss upon decomposition of the samples which took place at a high temperature above melting points with a decomposition and/or evaporation phenomenon at ~180 and 198°C for INHGA (2:1), and PGA (2:1), respectively (Figures 3A,B). A total mass loss of 92.0 and 98.2% was recorded for the respective co-crystals.

**Figure 4:**
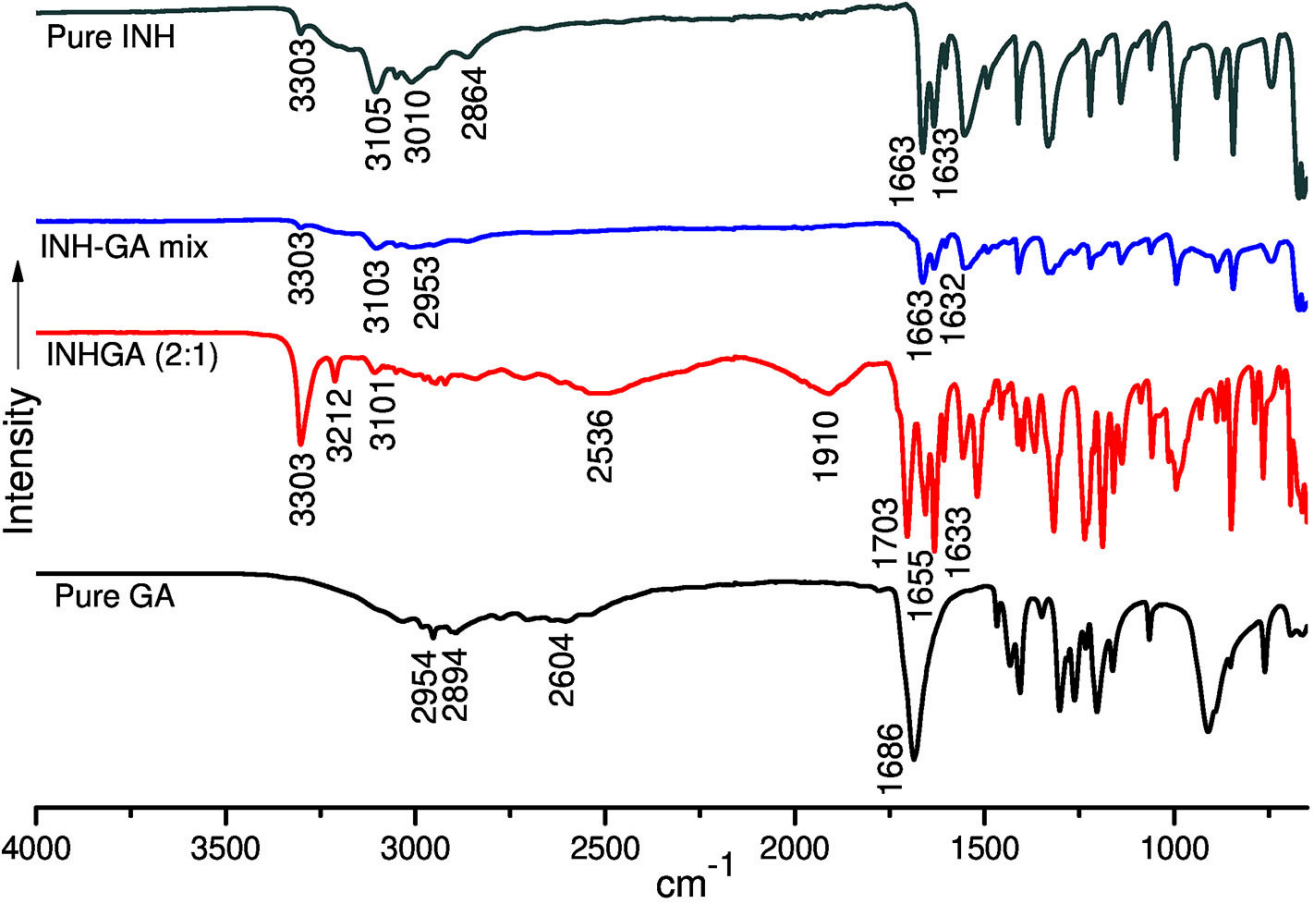
The FTIR spectra of isoniazid (INH), pentanedioic acid (GA), and INH-GA as a physical mixture and the (2:1) co-crystal.

**Figure 5:**
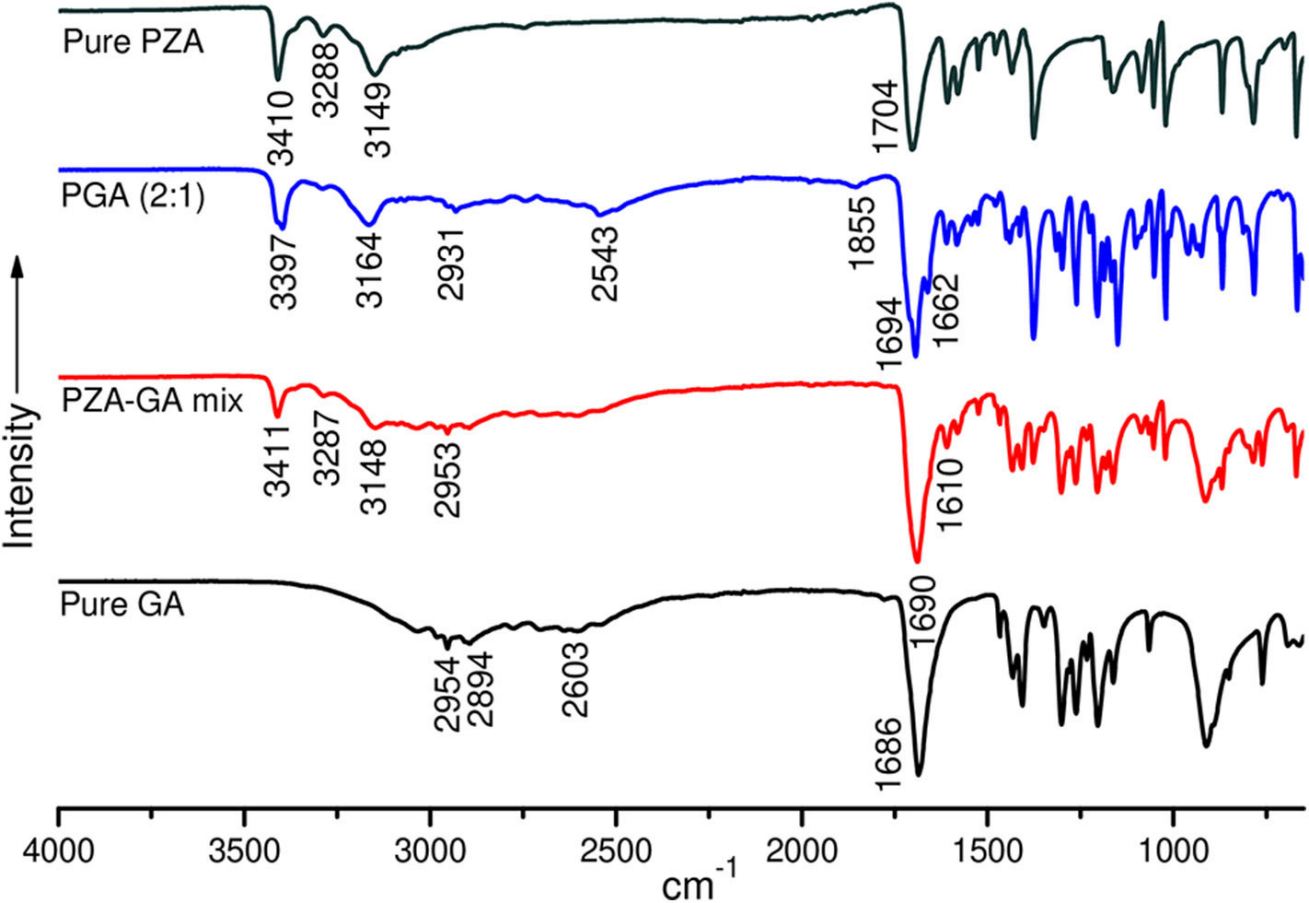
FTIR spectra of pure pyrazinamide (PZA), PGA (2:1) co-crystal, 2:1 physical mixture, and pure pentanedioic acid (GA).

Structural studies by FTIR were conducted to confirm the formation of the co-crystals by monitoring spectral changes of band positions in the spectra of INHGA (2:1) and PGA (2:1) co-crystal samples in comparison to pure INH and PZA and co-former GA. Such changes only occur as a result of intermolecular interaction between co-formers and confirm if a co-crystal was formed (Ali et al., 2012; Kumar, 2017). Figures 4, 5 illustrate IR spectra of INH, PZA, GA, and both INHGA (2:1) and PGA (2:1) co-crystals.

FTIR spectra of the individual drugs INH, PZA, and GA were obtained first and were used as references to analyze the co-crystal spectra. The INH spectrum exhibits two stretching amide ν(N-H) bands at 3,303 and 3,107 cm^−1^, stretch ν(C-H) at 3,010 cm^−1^, while amide stretching carbonyl at 1,663 cm^−1^ and C=C at 1,633 cm^−1^ in agreement with the literature (Gunasekaran et al., 2009; Ravikumar et al., 2013). The FTIR spectrum generated by GA is mainly characterized by a series of hydroxyl O-H stretching bands at 2,954, 2,894, and 2,604 cm^−1^ and the stretching carbonyl C=O at 1,686 cm^−1^.

The analysis of the IR spectrum of INHGA (2:1) co-crystal indicated that the INH carbonyl ν(C=O) stretch band shifted to 1,656 cm^−1^ while the GA stretching carbonyl ν(C=O) at 1,686 cm^−1^ shifted to 1,704 cm^−1^ due to H-bonding N-H^…^C=O. Further, less intensified GA carboxyl ν(O-H) at 2,604 cm^−1^ significantly shifted to 2,536 cm^−1^ as a result of H-bonding to N-H of INH. A new broad absorption peak at 1,910 cm^−1^ in the INHGA (2:1) spectrum further indicated the H-bonding effect. Other shifts identified are illustrated in Table 2.

**Table 2:** Summary of the different band shifting in the spectra of the INHGA (2:1) and their functional groups.

**Functional groups**	**INH**	**INHGA (2:1)**	**GA**	**Comments**
Stretching N–H	3,303 cm^−1^	3,303 cm^−1^	–	Increased intensity in INHGA (2:1)
Asym C–H	3,107 cm^−1^	3,101 cm^−1^	–	Shifting of C-H in INHGA (2:1)
Stretching O–H	–	–	2,954 cm^−1^	O-H band subsided in the INHGA (2:1)
C–H	3,010 cm^−1^	–	–	Disbanded in INHGA (2:1)
O–H	–	2,536 cm^−1^	2,894 cm^−1^ 2,604 cm^−1^	The band shifted and less intensified
	–	1,910 cm^−1^		A new band in the INHGA (2:1)
Carbonyl C=O	1,663 cm^−1^	1,704 cm^−1^	1,683 cm^−1^	Shifting in INHGA (2:1)
Stretching C=N	–	1,633 cm^−1^	1,632 cm^−1^	The band is more intensified in INHGA (2:1)

The FTIR spectra of pure PZA, GA, and PGA (2:1) co-crystal samples are presented in Figure 5 and Table 3. The significant differences in the N–H, O-H, and C=O stretching vibrations confirmed the generation of the indistinguishable co-crystal.

**Table 3:** Summary of the different band shifting in the spectra of PGA (2:1) and their assignments.

**Functional groups**	**PZA**	**PGA (2:1)**	**GA**	**Comments**
N–H	3,409 cm^−1^	3,398 cm^−1^		Broadening and shift in PGA (2:1)
Sym-N-H	3,288 cm^−1^			Subsided
Stretching C–H	3,148 cm^−1^	3,159 cm^−1^		Bands shifted in PGA (2:1)
Carboxylic O–H		2,931 cm^−1^ 2,543 cm^−1^	2,954 cm^−1^ 2,604 cm^−1^	Band shifted significantly
Carbonyl C=O	1,704 cm^−1^	1,694 cm^−1^		Significant band shifting
Carbonyl C=O		1,662 cm^−1^	1,686 cm^−1^	Significant band shifting

The PZA IR spectrum exhibited characteristic bands at 3,410, 3,288, and 3,149 cm^−1^, respectively, corresponding to asymmetric and symmetric amides stretching ν(N-H) and ν(=C-H) stretching. The absorption band at 1,704 cm^−1^ is assigned to stretching carbonyl ν(C=O) (Chiş et al., 2005; Gunasekaran et al., 2009).

Displacement of the PZA asymmetric stretching ν(N-H) band to 3,398 cm^−1^ and subsidence of the symmetric ν(N-H) stretching band in the spectra of PGA (2:1) co-crystal samples confirmed the contribution of amide N-H in H-bonding with GA carbonyl. The shifting of both PZA and GA carbonyl stretching ν(C=O) to 1,699 and 1,662 cm^−1^ positions in PGA, respectively, further suggested C=O^…^N-H bonding. The displacement of GA ν(O-H) stretching vibration at 2,954/2,604 to 2,931/2,543 cm^−1^ in the spectrum of PGA (2:1) co-crystal suggested O-H^…^N-H H-bonding to PZA amide ν(N-H).

**Figure 6:**
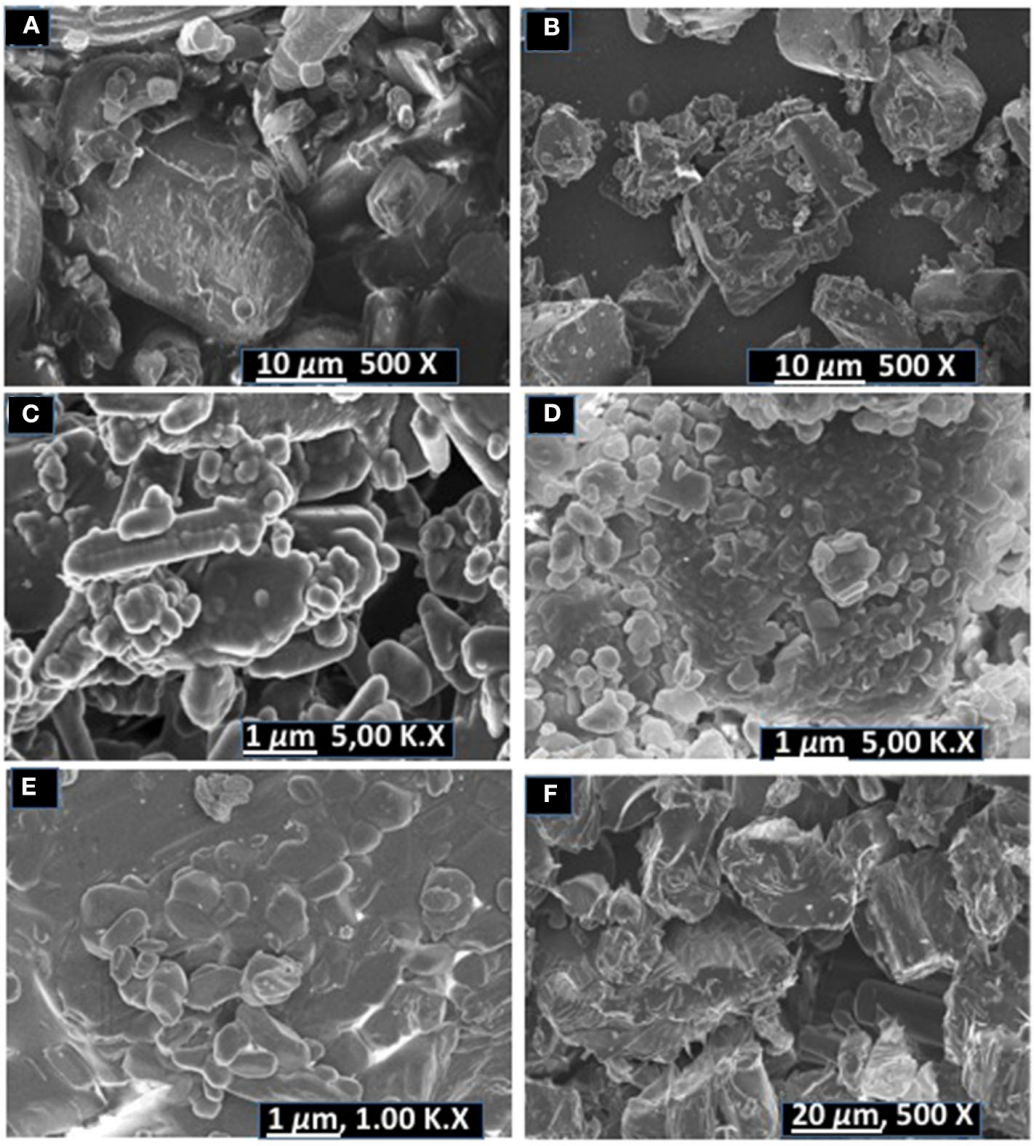
SEM micrographs of **(A)** pyrazinamide, **(B)** isoniazid, **(C)** PGA (2:1) co-crystal, **(D)** INHGA (2:1) co-crystal **(E)** pentanedioic acid, and **(F)** isoniazid-pentanedioic acid physical mixture.

SEM analysis was carried out to evaluate changes in the morphology of the co-crystal samples vs. pure untreated components. SEM micrographs are shown in Figure 6.

INH exhibited dusty irregularly shaped particles with sharp edges. GA exhibited round shapes and also have smooth surfaces. The INHGA (2:1) (Figure 6D) co-crystal exhibited a mixture of fragmented hexagonal and irregularly shaped particles with sharp surfaces, different from both INH (Figure 6B) and GA, and dusty aggregates due to the grinding exercise. The physical mixture sample is composed of the short-range order of particles from INH and GA (Figure 6F).

The SEM images of untreated PZA, GA, and PGA (2:1) are presented in Figures 6A,C,E. Pure PZA powder is composed of large and differently shaped crystals whereas PGA (2:1) powder from dry grinding is made of small amassed aggregates of round shapes (see supporting information). Rod-like particles mixed with round irregularly shaped aggregates with smooth edges were observed for the PGA (2:1) co-crystal sample obtained from solvent assisted grinding.

**Figure 7:**
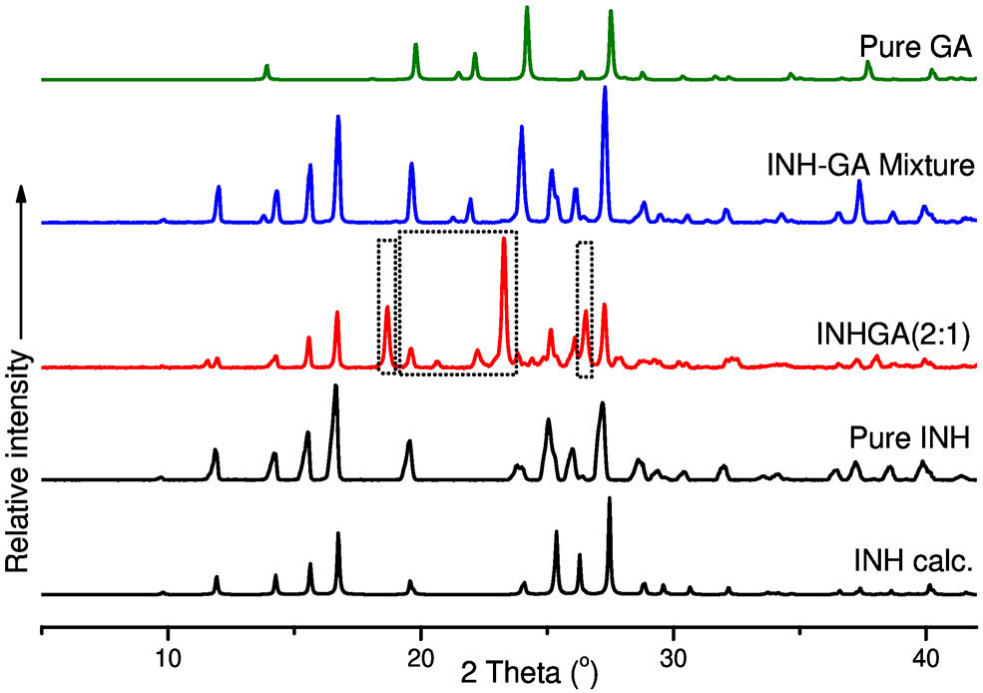
PXRD patterns of pure and calculated isoniazid, pentanedioic acid, INHGA (2:1) co-crystal, and INH-GA physical mixture.

**Figure 8:**
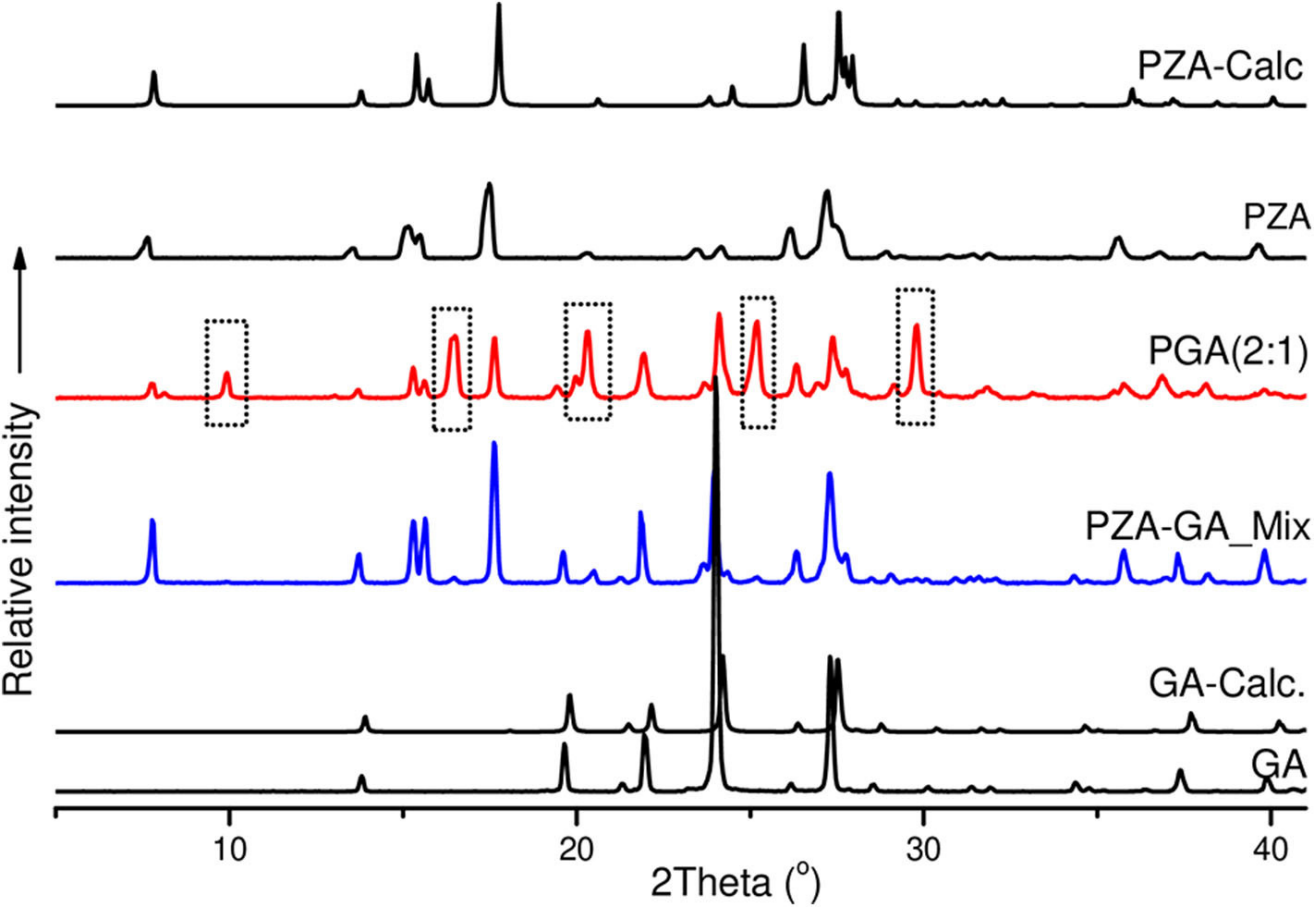
PXRD patterns of pure and calculated pyrazinamide, pure and calculated pentanedioic acid, PZA-GA physical mixture and PGA (2:1) co-crystal.

The co-crystals were further confirmed by PXRD analyses. In the absence of single X-ray diffraction data for the structure elucidation, PXRD is a non-destructive tool to confirm the new phases of the powder samples (Corvis et al., 2010; Padrela et al., 2012). PXRD authenticated the successful co-crystallization as previously confirmed by FTIR and other thermal analyses used. The patterns for the individual drugs and the co-crystal products are depicted in Figures 7, 8.

Differences in patterns is a clear confirmation of the successful co-crystal formation. The INH PXRD pattern exhibits peaks at 2θ = 11.9, 14.3, 15.6, 16.7, 19.6, 25.3, 26.9, and 27.3°. The main diffraction peaks in GA pattern appear at 2θ = 13.8, 19.7, 22.2, and 28.0°. The INHGA (2:1) PXRD pattern exhibited new diffraction peaks at 2θ = 18.7, 2, and 27.4° (Figure 7), thus confirming the new phase and the successful co-crystal formation.

The PZA PXRD pattern showed peaks at 2θ = 7.7, 15.5, 17.6, 27.0, and 27.3° whereas the pattern generated by co-crystal sample PGA (2:1) exhibited new peaks at 2θ = 10, 16.5, 20.4, 25.2, and 29.8° (Figure 8) and was, therefore, confirmed as a new phase.

*In vitro* solubility measurements were obtained using HPLC. After the necessary dilutions, samples were analyzed using HPLC at optimum wavelength of 262 and 270 nm detection for INH and PZA, respectively. The calibration curves for the two individual drugs INH and PZA were reported with a regression-squared value of 0.9997 and 0.09999, respectively (Supplementary Figure 3).

An increase in solubility of 1.7 and 1.3 times, was measured at acidic pH values 1.2 (0.1 N HCl) and 5.8 (PBS) for INHGA (2:1) co-crystal. At pH 7.4, the solubility increase was less significant (1.03-times increase) whereas the aqueous solubility of the co-crystal had slightly decreased. This suggested the influence of pH on the solubility of the co-crystal and the pure INH.

**Figure 9:**
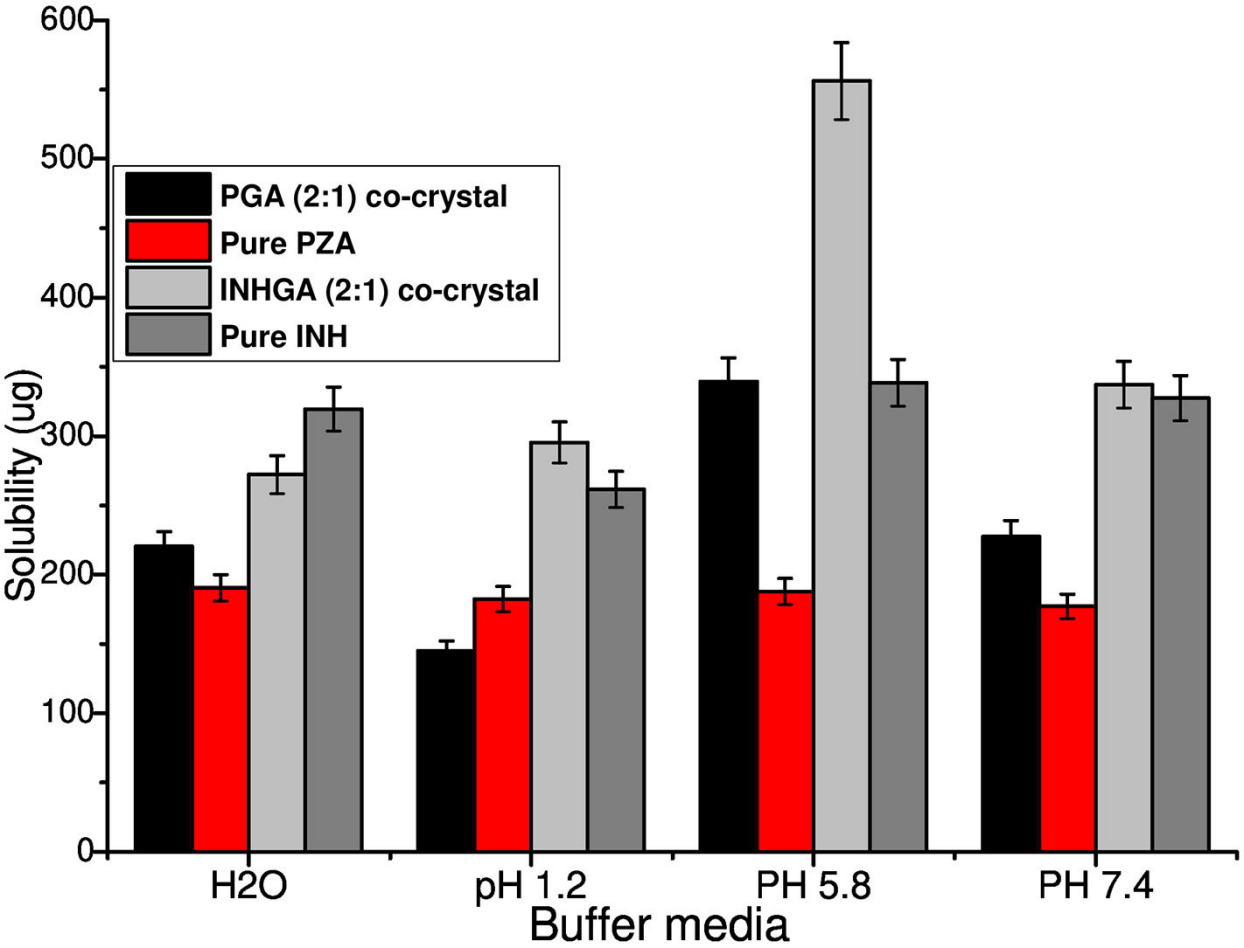
Histogram comparing the solubility of untreated isoniazid (INH) and pyrazinamide (PZA) to their respective co-crystals INHGA (2:1) and PGA (2:1).

PGA (2:1) on the other hand, has shown a solubility of 1.5, 1.8, and 1.3-times higher than those recorded for pure PZA, respectively, in the aqueous medium, potassium phosphate buffer at pH 5.8, and 7.4 (Figure 9). Despite an increase in solubility of PGA (2:1) co-crystal in aqueous solution, the co-crystal exhibited reduced solubility in acidic medium pH 1,2 (0.1 N HCl), suggesting increased stability of PZA by co-crystallization. The solubility of the co-crystals may be increased or decreased depending on the crystal density and strength of the hydrogen bond synthons. The higher the crystal packing, the more stable a co-crystal is and the less soluble it becomes (Rajesh Goud et al., 2014; Sugandha et al., 2014). Even though no pH-dependence was observed in pure PZA solubility measurements, its co-crystal with glutaric acid PGA suggested otherwise. The observed increase of solubility may further be attributed to reduced melting points of co-crystals in comparison to the pure APIs.

## Conclusion

Co-crystals INHGA (2:1) and PGA (2:1) co-crystal were successfully prepared using co-grinding; a simple, safe, and cost-effective method. Characterization established different properties signifying both to be novel co-crystals. Solubility studies conducted suggested an enhanced solubility of both drugs as co-crystals, in different pH environments. The co-crystal formation is known to stabilize unstable drugs (Ranjit and Sarma, 2018), therefore reduced solubility of the co-crystal INHGA (2:1) in the aqueous medium is plausibly due to an increased stability. Notably, increased solubility and stability of the co-crystals vs. individual APIs infer what pharmaceutical formulators desire.

## Data Availability Statement

All datasets generated for this study are included in the article/Supplementary Material.

## Author Contributions

JN: methodology, validation, formal analysis, investigation and data curation, writing original draft, visualization, and writing—review and editing. MA: resources and data curation and writing—review and editing. PP: resources, data curation, and writing—review and editing. HS: conceptualization, resources, writing—review and editing, supervision, project administration, and funding acquisition. All authors contributed to the article and approved the submitted version.

## Conflict of Interest

The authors declare that the research was conducted in the absence of any commercial or financial relationships that could be construed as a potential conflict of interest.
