# Supplementary material for: Mechanochemical Synthesis and Physicochemical Characterization of Isoniazid and Pyrazinamide Co-crystals With Glutaric Acid
Source: Front Chem. 2020 Nov 16;8:595908. doi: 10.3389/fchem.2020.595908 (PMC7706006; doi:10.3389/fchem.2020.595908)
Supplement: Supplementary file 1 [file Data_Sheet_1.doc]

Supplementary Figure 1: Molecular structures of (A) isoniazid (INH) (B) pyrazinamide (PZA), and (C) pentanedioic acid (GA)(NB: DR= dry grinding, LAG= liquid assisted grinding, and SE= solvent evaporation)


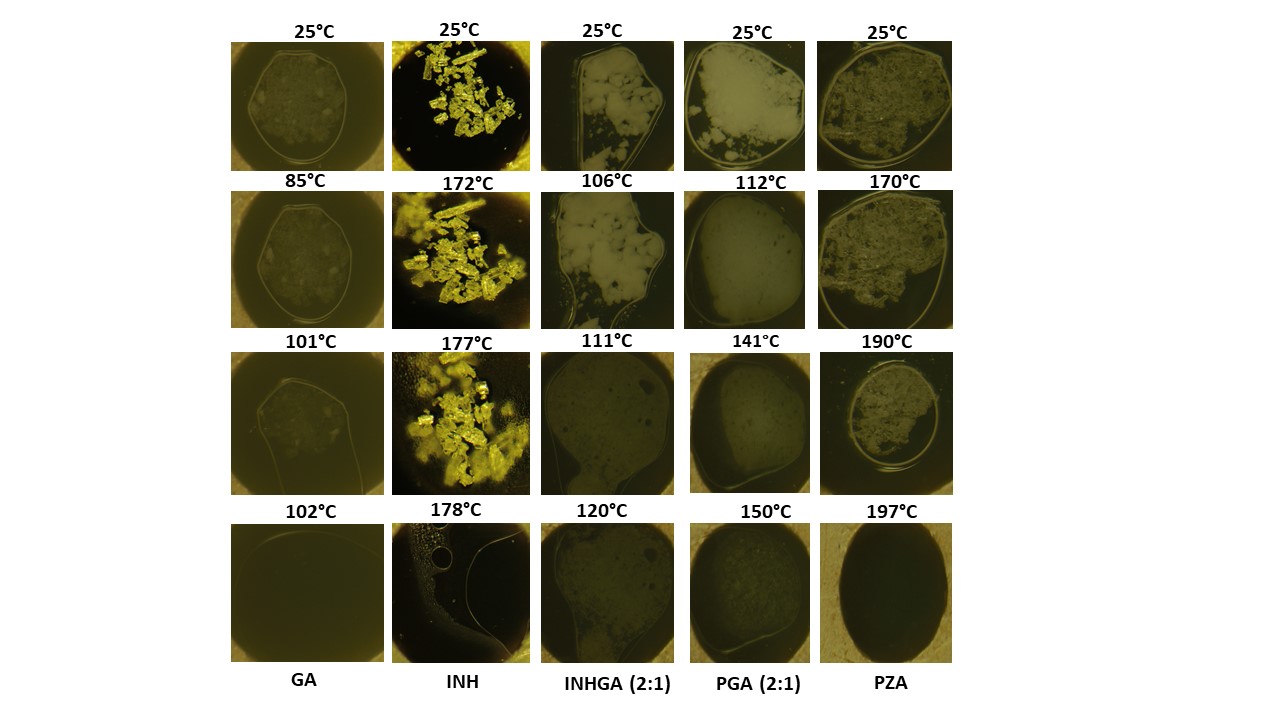


Supplementary Figure 2: HSM images of pentanedioic acid (GA), isoniazid (INH), the co-crystals INHGA (2:1), PGA (2:1) and pyrazinamide (PZA) recorded over 25-200 °C temperature range.


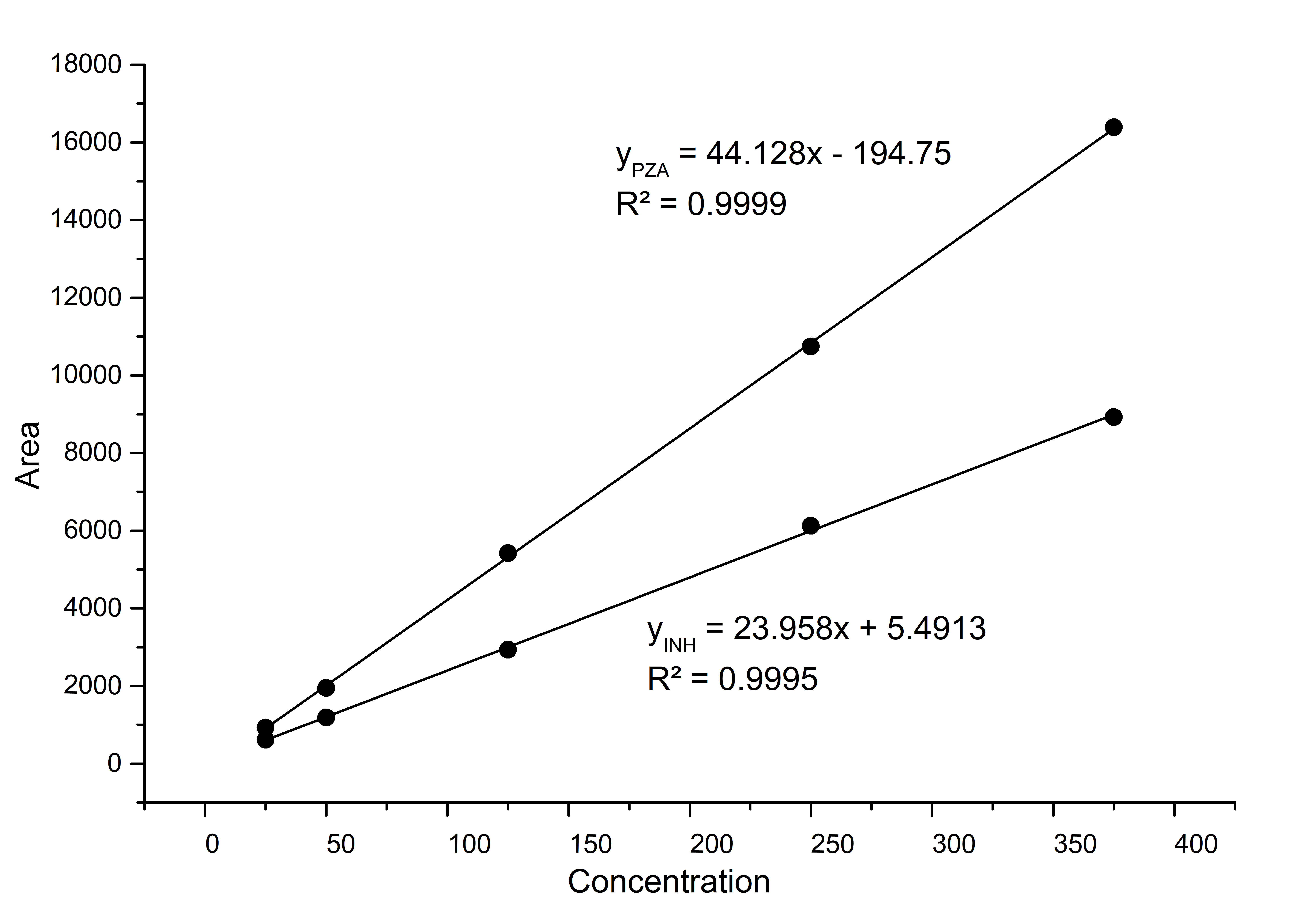


Supplementary Figure 3: Standard calibration curves for isoniazid (INH) and pyrazinamide (PZA) for INHGA (2:1), PGA (2:1) co-crystals.
